# New insights into the microbiota of wild mice

**DOI:** 10.1007/s00335-021-09887-z

**Published:** 2021-07-09

**Authors:** Ho-Keun Kwon, Je Kyung Seong

**Affiliations:** 1 Department of Microbiology and Immunology, Institute for Immunology and Immunological Diseases and Brain Korea 21 PLUS Project for Medical Sciences, Yonsei University College of Medicine, Seoul, Korea; 2 Laboratory of Developmental Biology and Genomics, Research Institute for Veterinary Science, and BK 21 PLUS Program for Creative Veterinary Science Research, College of Veterinary Medicine, Seoul National University, Seoul, 08826 South Korea; 3 Interdisciplinary Program for Bioinformatics, Program for Cancer Biology and BIO-MAX/N-Bio Institute, Seoul National University, Seoul, 08826 South Korea; 4 Korea Mouse Phenotyping Center (KMPC), Seoul National University, Seoul, 08826 South Korea

## Abstract

Laboratory mice have long been an invaluable tool in biomedical science and have made significant contributions in research into life-threatening diseases. However, the translation of research results from mice to humans often proves difficult due to the incomplete nature of laboratory animal-based research. Hence, there is increasing demand for complementary methods or alternatives to laboratory mice that can better mimic human physiological traits and potentially bridge the translational research gap. Under these circumstances, the natural/naturalized mice including “wild”, “dirty”, “wildling”, and “wilded” systems have been found to better reflect some aspects of human pathophysiology. Here, we discuss the pros and cons of the laboratory mouse system and contemplate how wild mice and wild microbiota are able to help in refining such systems to better mimic the real-world situation and contribute to more productive translational research.

Laboratory mice (lab mice) are a mainstay in current biomedical science that have contributed to pivotal discoveries leading to the development of life-saving therapeutics (Kleinert et al. 2018; Rust 1982). The main advantage of using lab mice is the ability to control genetic and environmental factors, which are key determinants for the experimental outcome of biomedical research. The use of lab mice, mainly comprising a wide range of inbred strains, enables researchers to control and even manipulate their genetic components, permitting the elucidation of the causal relationship between the gene and the specific phenotype of cells and/or animals. At the same time, the standardized environmental conditions in the present-day animal vivarium are tightly controlled and have led to synchronization of the majority of environmental factors (for example, day/night cycle, temperature, diet, and water supplementation, etc.), which potentially affects the experimental outcome. (Reza Khorramizadeh and Saadat 2020). Moreover, lab mice can be maintained in an artificial microbial environment, known as specific pathogen free (SPF), in which the exposure of lab mice to the major pathosymbiotics (bacteria, viruses, fungi, and worms) is highly restricted compared with that of mice in a natural habitat (Nguyen et al. 2015). These advantages of the laboratory mouse system have drastically enhanced the reproducibility of biomedical research and have allowed researchers to leverage a “one gene–one disease” paradigm.

However, there is growing concern for the validity of laboratory animal models that do not properly manifest human pathophysiological traits (Mak et al. 2014; Pedersen and Babayan 2011; Seok et al. 2013). For example, despite the astronomical costs of animal model-based preclinical research, the overall success rate of subsequent drug development trials is just 14% (Wong et al. 2019). Furthermore, mouse and human studies have occasionally come to contrasting conclusions, which has cast doubt on the effectiveness of lab mice in translational research (Fisher et al. 1996; Naqvi et al. 2019; Puellmann et al. 2006; Seok et al. 2013). Meanwhile, recent studies have indicated that genetic and environmental regularity in lab mice may be a potential cause of these discrepancies (Churchill et al. 2004; Viney et al. 2015). For instance, the majority of current immunological knowledge was primarily gleaned from a few inbred mouse strains, thereby limiting the amount of noise caused by segregating genetic variations. Thus, much of our biomedical knowledge, is built on a very small number of individual mice; this would correspond to only 5 to 10 human individuals, which would hardly be enough to emulate the complex genetic diversity and vulnerability of human beings (Pedersen and Babayah 2011). Moreover, together with genetic factors, the environmental variables of age, socioeconomic status, climate, nutritional status, health status, etc.—none of which can be accounted for in lab mice—have also been shown to affect human immune responses (Brodin et al. 2015; Rohr et al. 2011). Above all other environmental influencers, it has recently become clear that microbial communities, known as the microbiota, play an invaluable role in the physiology of hosts. Microbiota have been shown to influence processes from organ development/morphogenesis and metabolism to the development, differentiation, and function of host immunity (Belkaid and Hand 2014; Sommer and Bäckhed 2013). Indeed, germ-free (GF) mice, unexposed to microorganisms, have been found to exhibit a broad spectrum of developmental and functional impairment in the immune system, directly linked to susceptibility to various immunological disorders including cancer, allergies, inflammatory bowel disease (IBD), and autoimmune diseases (Round and Mazmanian 2009). There is therefore increasing demand in various areas of biomedical research to develop an alternative and/or complementary system for lab mice that can minimize the chasm between basic and translational research (Masopust et al. 2017; von Scheidt et al. 2017).

The study of wild mice seems to be an alluring alternative with which to compensate for the limited translational value of lab mice (Poh 2019; Nobs and Elinav 2019). In contrast to lab mice, wild or free-living mice exhibit preserved genetic diversity as well as environmental effects, which, as stated above, are the key determinants of human ecophysiology. Despite the extreme lack of research involving wild mice, previous studies have pinpointed human-like immunological traits in such mice—for example, a ‘primed’ immune state and its potential link with the cumulative exposure to microbes in nature—compared with mice living in a laboratory vivarium (Table 1) (Abolins et al. 2017, 2011; Beura et al. 2016; Boysen et al. 2011; Lochmiller et al. 1991). Indeed, a recent study by Lalit and colleagues showed that immune systems in lab mice have phenocopied a human neonate-like immune status, compared with those of wild mice, in which human adult-like immune traits were observed (Beura et al. 2016). The unique immunological characteristics of feral mice are highly reminiscent of human immune traits, suggesting that wild mouse systems may be a potential tool to bridge the gap between lab mice and translational research. Studies have shown that wild mice have a complex and diverse microbiome, including a mycobiome, archaeome, and parasitome, similar to those found in humans (Table 2) (Beura et al. 2016; Lavrinienko et al. 2018; Linnenbrink et al. 2013; Rosshart et al. 2019, 2017; Song et al. 2021; Suzuki and Nachman 2016; Weldon et al. 2015; Williams et al. 2018). As the microbiome is known to configure host physiology and pathophysiology, the higher orders of the microbial communities in wild mice, shaped by natural selection in the wild, might play distinct roles in host fitness. For example, primed immune traits in wild mice have shown a strong association with age and infectious burden with remarkable heterogeneity (Abolins et al. 2017; Beura et al. 2016), indicating that cumulative microbial exposure may be one of the key drivers of immune activation/maturation in wild mice, in a manner similar to that seen in humans (Salgame et al. 2013; Virgin et al. 2009). Furthermore, the natural gut microbiota has been shown to improve the outcome of viral infection and tumorigenesis in lab mice (Rosshart et al. 2017). Although it would be somewhat premature to discuss the validity of the use of wild mice in current biomedical research, as very little research has yet been carried out, the studies cited above collectively highlight the potential of the wild mouse system as a tool in translational research that can better mimic the biological evolution of humans and diseases.

**Table 1:** Distinct features of the wild immune system

Numbers	Species	Source of animals	Tissues	Major findings	Assays	References
1	*Peromyscus leucopus, Microtus pinetorum, Perognathus hispidus, Neotoma Jloriakna, Onychomys leucogaster, Mus musculus*	Central Oklahoma (USA)	Blood	-Increased primary immunoresponsiveness to SRBC	Splenic plaque-forming cell assay	Lochmiller RL et al. (1991)
2	*Mus musculus*	Bristol (UK)	Blood, spleen	-Enhanced antibody production against keyhole limpet hemocyanin (KLH) immunization -Overall activation status of immune cells (T cells, B cells, DCs, and Macrophage)	KLH immunization and flow cytometric (FACs) analysis	Abolins SR et al. (2011)
3	*Mus musculus*	southeastern Norway	Spleen	-Primed status of NK cells (Increase of CD69, KLRG1, GRAZYME B, IFNγ and NKp46 expression, and CD27^+^CD11b^−^population)	FACs analysis	Boysen P et al. (2011)
4	*Mus musculus*	Minnesota and Georgia (USA)	Blood, spleen	-Primed status of CD8^+^ T cells (increase of CD44^+^CD62L^−^ T cells)	Serological, FACs, and RNAseq analysis	Beura LK et al. (2016)
5	*Mus musculus*	Bristol and Stroud (UK)	Blood, spleen	-Higher level of serum Proteins (IgG, IgE, SAP, Haptoglobin) -Highly primed state of overall immune population (CD44^+^CD62^−^ T cells, CD27^+^CD11b^−^ NK cells)	Serological and FACs analysis	Abolins S et al. (2017)
6	*Mus musculus*	Maryland and Columbia (USA)	Blood, spleen, liver, vagina, skin, and gut	-Enrichment of immune responses/activation signature in PBMC	CyTOF and RNAseq analysis	Lavrinienko A et al. (2018)

**Table 2:** Distinct features of the wild microbiome

Numbers	Species	Source of animals	Source of bacteria	Major findings	Assays	Reference
1	*Mus musculus*	Cologne/Bonn and Schömberg/Langenbrand (Germany) Severac le Château, Espelette, Angers, Nancy, Louan‐Villegruis and Divonne les Bains (France)	Gut	Dominant genera (Bacteroides, Robinsoniella, and Helicobacter) Geography: key determining factor for the patterns of microbial diversity	16S rRNA sequencing	Linnenbrink M et al. (2013)
2	*Mus musculus*	Gloucestershire, Bristol, London (UK)	Gut	Geography: the key determining factor for microbial diversity and composition Correlation of microbial diversity with host's weight, leptin, parasite, and virus level	16S rRNA sequencing	Weldon L et al. (2015)
3	*Mus musculus*	Tucson (USA)	GI tract contents	Different microbial composition between upper and lower GI tract	16S rRNA sequencing, Spatial bacteria composition analysis along GI tract	Suzuki and Nachman (2016)
4	*Mus musculus*	Minnesota and Georgia (USA)	Gut	Immune prime by co-housing in inbred mice High level of viral/fungal/worm infection in wild/pet-store mice	Infectious agent screening, Co-housing	Beura LK et al. (2016)
5	*Mus musculus*	Maryland and Columbia (USA)	Gut	Higher diversity/complexity of wild microbiome Enhancing host fitness to Influenza infection and colitis-associated tumorigenesis	Shotgun metagenome sequencing, 16S rRNA sequencing, FMT	Rosshart SP et al. (2017)
6	*Mus musculus*	New York City (USA)	Gut	Carrying gastrointestinal disease-causing microbials (Shigella, Salmonella, Clostridium difficile, and diarrheagenic Escherichia coli) Enrichment of antimicrobial resistance genes	16S rRNA sequencing, antimicrobial resistance test	Williams SH et al. (2018)
7	*Myodes glareolus*	Chernobyl Exclusion Zone and Kyiv (Ukraine)	Gut and skin	Geography: key determining factor for skin microbiome Radioactive contamination: key determining factor for gut microbiome	16S rRNA sequencing	Lavrinienko A et al. (2018)
8	*Mus musculus*	Maryland and Columbia (USA)	Gut, skin, and vagina	Higher diversity/complexity (virome and mycobiome) Resilience to environmental challenge (high fat diet, antibiotics treatment, co-housing) Assigning human-like traits (CD28 super-agonist or anti-TNFα treatment)	Shotgun metagenomic sequencing, 16S rRNA sequencing, Mycobiome sequencing, Virome sequencing, Pathogen screening, High fat diet, antibiotics treatment, and co-housing	Rosshart SP et al. (2019)
9	*Micromys minutus* and *Mus musculus*	Chuncheon (Korea)	Gut	Different microbial composition between *M. minutus* and *M. musculus* High prevalence of Campylobacter in microbiota of *M. minutus*	16S rRNA sequencing	Song H et al. (2021)

Human beings have co-evolved alongside trillions of microbes, considered to be a “hidden organ” due to their immense impact on human health and disease (Cho and Blaser 2012; O'Hara and Shanahan 2006; Pflughoeft and Versalovic 2012). Recent advances in microbiome research have outlined the indispensable roles of microbiota in the induction, training, and function of the host immune system in both humans as well as rodents (Belkaid and Hand 2014), and microbial exposure to acute and chronic pathogens has been shown to control immune variation in humans (Salgame et al. 2013; Virgin et al. 2009). In addition, individuals with a heavy helminth burden have been found to show compromised vaccination-induced immune responses with Bacillus Calmette-Guerin (BCG) and cholera (Cooper et al. 2001; Elias et al. 2001). Meanwhile, a number of studies have shown the versatility of microbiota in modulating host physiology and functions in rodent (Belkaid and Hand 2014; Round and Mazmanian 2009; Sommer and Bäckhed 2013; Surana and Kasper 2014). However, as the majority of lab mice are raised in an SPF vivarium, as a result of which they have extremely simple and controlled microbiota, the lack of microbial diversity and complexity in lab mice might be an environmental deficit that decreases the value of lab mice to human translational research. Indeed, recent studies have exquisitely demonstrated how the “dirty” microbiota from wild mice can be used as a tool to recapitulate naturally selected microbial niches to bolster the translational validity of lab mice (Beura et al. 2016; Rosshart et al. 2019, 2017). Lalit and colleagues were the first to show the potential of dirty microbiota as a valuable tool in lab mouse-based immunology by co-housing pet-store mice with lab mice (Beura et al. 2016). Intriguingly, co-housing with pet-store mice was sufficient to increase the differentiated effector memory T cell population (CD44^hi^), which produces high levels of *Granzyme B*, in the peripheral immune system of the lab mice. Moreover, the authors found *de novo* populating CD8^+^ T cells, which have a tissue-resident memory T cell (T_RM_) phenotype, in non-lymphoid organs of co-housed C57BL/6 mice, similar to those seen in human tissues. Finally, the transcriptional signatures in blood cells from the lab mice that were co-housed with pet-store mice were found to have reshaped signature patterns that more closely mirrored adult than neonatal ones. Altogether, these studies highlight that, beyond genetic elements, microbiota play a key role in determining basal immunological states, and suggest that dirty microbiota may be of use in restoring physiological microbial states and provoking immunological traits observed in human beings. Indeed, another study discovered that sequential infection with defined pathogens including herpesvirus, influenza, and helminth in lab mice elicited altered transcriptome signatures that partially recapitulated the immunological features triggered by dirty microbiota (Reese et al. 2016). This suggests that selective microbial exposure (termed “defined dirty”) can be used to install human-like immunological traits in lab mice.

To study the importance of host–microbe interaction on host physiology, Rehermann’s group at NIH has trapped more than 800 wild mice from eight different locations, characterized their gut microbiota and compared them with those of lab mice (Rosshart et al. 2017). As expected, wild mice exhibited more diverse and complex microbiome compositions with well-preserved natural features, including viral and fungal species, compared to those of lab mice. Interestingly, the wild microbiome can be transferred to, and maintained in, lab mice over several generations (up 5^th^ generation) without notable changes, suggesting that there is relatively minor impact of other environmental factors, including temperature, day/night cycle, diet, and social structure, on the microbiome community (Rosshart et al. 2019, 2017). Similar to these results, we have documented the resilience and stability of the wild microbiome in inbred mice up to the 15th generation (unpublished data), strengthening the potential value of “dirty” microbiota in translational research.

The potential benefits of the wild microbiome to the fitness of lab mice have been evaluated in a disease context. Following influenza infection, compared with laboratory mice (lab) or laboratory microbiome-transferred mice (labR), wild microbiome-implanted mice (wildR) showed significantly decreased mortality (lab/labR: 83% vs. wildR: 8%), viral burden, and lung pathology. Additionally, researchers have found that fecal microbiota transplantation (FMT) of wild mouse gut microbiota conferred protection against inflammation-induced neoplastic development. Altogether, these results suggest that the “natural microbiota” (microbiota from free-living mice and humans) have co-evolved to promote host fitness and survival under natural selection pressure, to establish a symbiotic host–microbe interaction that is integral to host physiology, particularly in the case of the immune system. To take “natural microbiota” engraftment a step further, the same group devised a model in which they transferred C57BL/6 embryos into a pseudo-pregnant dam, captured in the wild (Rosshart et al. 2019). The pups from the wild dams, called “wildlings”, were shown to retain much of the wild microbiota via vertical transmission from the wild dam, but with the less complex genetics of C57BL/6. In a manner similar to that of the natural microbiota engraftment system, wildlings have well-retained naturally derived microbiome compositions, such as those of the mycobiome and virome, that can be vertically transferred to their offspring for multiple generations without notable diversification, indicating the resilience of the natural microbiome. Next, they comprehensively profiled and compared the immune states of lab, wild, and wildling mice to estimate the contribution of genetics (wild vs. wildling) or microbiome (lab vs. wildling) on the host immune system by cytometry, using time of flight (CyTOF) and RNA sequencing-based transcriptome analysis (RNAseq). They found more similar transcriptome patterns of blood mononuclear cells from wildlings when compared with wild mice than lab mice, despite their genetic differences. Intriguingly, these researchers have shown the considerable influence of the microbiome on immune cells in central lymphoid tissue but not in non-lymphoid tissue, which indicates that genetics and microbial composition combine to influence host immunity. Finally, the translational value of wilding was evaluated by the retrospective validation of two well-known models, CD28-super-agonist (CD28SA) for the treatment of autoimmune disease, inflammatory disease, and transplantation (Puellmann et al. 2006) and anti-TNFα treatment for sepsis (Fisher et al. 1996), which have shown discordant results in lab mouse and human systems. Surprisingly, the treatment of CD28SA or anti-TNFα in wildlings, but not in lab mice, phenocopied the trait of immune responses observed in humans. Altogether, these results cast doubt on the validity of the current laboratory mouse system and suggest that wildlings may be a promising complement to lab mice for translational research in immunology.

In support of a wild/dirty/wildling system, Graham’s research group at Princeton University has recently built an outdoor mouse enclosure with eight wedge-shaped pens covering nearly 1,500m^2^, and has put lab mice in these “farms” to re-wild them in natural environments; such mice are termed “wilded”. This enables researchers to manipulate the key determinants in animal-based biomedical research, such as host genetics, age, and sex, while preserving naturally occurring environmental pressure (Graham 2021; Leung et al. 2018). Interestingly, re-wilding of lab mice has been found to partially phenocopy the ‘primed’ immune traits that exist in wild mice and humans (Leung et al. 2018; Yeung et al. 2020). Furthermore, a recent study has beautifully outlined the relative contributions of genetic versus environmental factors that govern inflammatory immune responses by re-wilding lab mice carrying *nod2* or *atg16l1* mutations, the well-known susceptibility mutations in inflammatory bowel disease (Lin et al. 2020). Overall, their results suggest that re-wilding lab mice could facilitate exploration of the effects of the natural environment in wildlife.

While laboratory animal-based research has led to notable advances in modern biomedical research, it has become apparent that lab mice are not always reliable in recapitulating human physiology and pathophysiology. This has led to significant demand for a novel platform that can mitigate the flaws or limitations of the lab mouse system. Accordingly, recent studies involving wild, dirty, wildling, and wilded systems have proposed these as potential complementary systems that maximize the translational validity of lab mice. Nevertheless, despite having significant congruence with human physiology, wild mice may not be technically easy to incorporate into general biomedical research facilities due to difficulties with animal supplementation and maintenance. As an alternative to the use of wild mice, FMT of natural microbiota from dirty, wildling, and wilded mice has been shown to elicit the recapitulation of major human pathophysiological traits in lab mice and is a promising tool of potential use in translational research. However, the application of these systems in current biomedical research poses the following difficulties: firstly, as a result of naturally occurring infections, these animals pose biosecurity and zoonotic issues that restrict their husbandry to facilities with biosafety level 2 or higher; furthermore, there is a huge difference in the overall composition of the microbiome depending on the source of wild mice, which may potentially lead to discordant research outcomes when using these systems. Thus, while these systems may not serve as a substitute for the use of lab mice in current biomedical research, they can be of use in enhancing the validity and translational power of animal-based preclinical research in human physiology and pathophysiology.
